# “Window of Opportunity” in Ocular Graft-Versus-Host Disease Treatment: Results of a Longitudinal Study and Case Reports

**DOI:** 10.1097/ICL.0000000000001081

**Published:** 2024-03-13

**Authors:** Bayasgalan Surenkhuu, Christine S. Mun, Christian Kim, Nour Yanna Atassi, Jessica Mun, Nikhil Dhall, Sarah Abdel-Hadi, Tanya Sheth, Priyanka Dondeti, Alexandria Bernal, Anubhav Pradeep, Damiano Rondelli, Sandeep Jain

**Affiliations:** Corneal Translational Biology Laboratory (B.S., C.S.M., C.K., N.Y.A., J.M., N.D., S.A.-H., T.S., P.D., A.B., A.P., S.J.), Department of Ophthalmology and Visual Sciences, University of Illinois at Chicago, Chicago, IL; and Department of Medicine (D.R.), Division of Hematology and Oncology, University of Illinois at Chicago, Chicago, IL.

**Keywords:** Dry eye, oGVHD, Window of opportunity, Early treatment

## Abstract

**Objective:**

To perform a longitudinal study for determining the development of ocular graft-versus-host disease (oGVHD) after allogeneic hematopoietic stem cell transplant (HSCT) and report cases that illustrate the “window of opportunity” concept in oGVHD treatment.

**Methods:**

Patients (n=61) were examined at prescheduled clinic visits before HSCT and three-month intervals after HSCT for 2 years. The presence or absence of oGVHD was determined using the international chronic oGVHD consensus group diagnostic criteria. Ocular surface washings (OSW) were obtained at each visit and analyzed for cytokine levels.

**Results:**

In the longitudinal study, 26.2% (n=16; progressed group) developed either probable (11.5%, n=7) or definite oGVHD (14.8%, n=9). In the progressed group, clinically significant changes in signs (corneal staining and Schirmer I test) and symptoms at the post-HSCT visit as compared with the pre-HSCT visit occurred at 9 months. Significant differences in clinical signs and symptoms (whether average post-HSCT values or changes in values over pre-HSCT levels) between the progressed and nonprogressed groups occurred at a 9-month visit or later. In the progressed group, 55.6% of eyes that had negative matrix metalloproteinase 9 (MMP-9) test at pre-HSCT turned MMP-9 positive at 3 to 6 months post-HSCT. In the progressed group, interleukin 8 levels in OSW were significantly increased at 6 months post-HSCT. In the case reports, the “window of opportunity” was detected by MMP-9 turning positive, early corneal staining, interleukin 8 increase in OSW, and peripheral corneal epithelial thinning, which resolved with treatment initiation.

**Conclusions:**

A “window of opportunity” exists before patients developing symptomatic tear-deficient dry eye after HSCT for initiating treatment that may preempt oGVHD development; however, larger-scale longitudinal studies are needed for definitive recommendations.

Chronic ocular graft-versus-host disease (oGVHD) is one of the most frequent and rapidly progressive organ manifestations with characteristic inflammatory, immune dysregulatory, and fibrotic pathophysiological mechanisms.^1,2^ The incidence of chronic oGVHD has been reported to be about 40% to 60%.^3^ Chronic oGVHD adversely affects quality of life.^4^ Therefore, early diagnosis and treatment of oGVHD may have significant clinical benefits. The absence or presence of chronic oGVHD is determined using two validated diagnostic classifications based on clinical signs and symptoms. The 2014 National Institutes of Health chronic GVHD diagnosis and staging consensus recommendations suggest diagnosing oGVHD based on a reduction in the Schirmer I test. Four severity scores (0–3) are based on the symptoms and frequency of artificial tear use.^5^ The International Chronic Ocular Graft-versus-Host Disease (ICCGVHD) consensus group diagnostic criteria are based on scores derived from the Ocular Surface Disease Index (OSDI), Schirmer I test without anesthesia, corneal fluorescein staining, conjunctival injection, and presence of systemic GVHD.^6^ Since both these classifications are based on the presence of clinical signs or symptoms of enough severity to make a diagnosis of oGVHD, it would follow that treatment initiation would be dependent on clinical signs and symptoms to manifest themselves in sufficient severity so that a diagnosis of oGVHD can be made.

Recently, the 2020 Clinical Implementation and Early Diagnosis Working Group Report of the National Institutes of Health Consensus Development Project on Criteria for Clinical Trials in Chronic Graft-versus-Host Disease recognized that many patients do not meet oGVHD diagnostic criteria until irreversible manifestations of the disease, such as sicca symptoms, have already developed.^7^ Therefore, the report recommended developing tools to recognize or predict the imminent onset of chronic oGVHD at an earlier stage before diagnostic criteria are met to allow preemptive interventions to prevent progression to irreversible damage.^7^ In this report, we present data from a longitudinal study and two case reports suggesting that early changes in tear fluid cytokines, corneal epithelial thickness, and intracorneal inflammatory cells may identify a “window of opportunity” for initiating early combinatorial treatment to preempt the progression to oGVHD.

## METHODS

Study approval was obtained from the Institutional Review Board of the University of Illinois at Chicago (UIC). Informed consent was obtained from all participants after the nature and possible consequences of the study were explained. Research was conducted in accordance with the tenets of the Declaration of Helsinki. Potential patients scheduled to receive HSCT in the subsequent 3 months were recruited via referral from physicians at the Departments of Hematology and Oncology at UIC, Northwestern University, and Rush University Medical Center.

All patients were evaluated at an initial baseline visit (pre-HSCT) and after HSCT, at 3-month intervals up to 24 months. Ophthalmological examination included (1) symptoms of ocular discomfort as measured by using the OSDI score. The OSDI questionnaire assesses the symptoms of ocular discomfort consistent with dry eye disease and their impact on vision-related functioning. The overall OSDI score defined the ocular surface as normal (0–12 points) or as having mild (13–22 points), moderate (23–32 points), or severe (33–100 points) disease,^8–10^ and (2) tear secretion as measured by using the Schirmer I test (without anesthesia over 5 min). The Schirmer I measurement ≤5 mm/5 min is considered severe tear fluid deficiency, and (3) the corneal staining score was measured by using Lissamine green dye staining using the National Eye Institute grading scale.^11^ The dye (5 μL of 1% solution) was applied to each eye and a slit lamp was used to observe corneal staining (×16 magnification, high illumination with a diffuser). The National Eye Institute scale relies on a chart that divides the cornea into five sections and assigns a value from 0 (absent) to 3 (severe) to each section, based on the density of punctate keratitis, for a maximum of 15 points. (4) The matrix metalloproteinase 9 (MMP-9) test was performed using the InflammaDry kit (RPS Diagnostics, Sarasota, FL). A negative test was scored as 0. A positive test was scored as 1.0 (faint positive), 2.0 (positive), or 3.0 (strong positive).

We used the ICCGVHD to diagnose oGVHD. These criteria included OSDI, the Schirmer I test without anesthesia, corneal staining, and conjunctival injection. In the presence of systemic GVHD, a score of 0 to 3 indicates the absence of ocular GVHD (none oGVHD), a score of 4 to 5 indicates probable ocular GVHD, and a score of ≥6 indicates definite ocular GVHD. In the absence of systemic GVHD, a score of 0 to 5 indicates the absence of ocular GVHD (none oGVHD), a score of 6 to 7 indicates probable ocular GVHD, and a score of ≥8 indicates definite ocular GVHD. The progression of oGVHD was defined as a score increase that would move an eye from none oGVHD to either probable or definite oGVHD.

### Ocular Surface Washings Collection and Analysis

Ocular surface washings was performed as described previously.^12^ Briefly, at the slit lamp, 50 μL of Preservative-Free artificial tears (Refresh Optive, Allergan, Madison, NJ) was instilled into the inferior fornix of the eye. After approximately 30 to 40 sec, OSW was collected from the inferior fornix with 10 μL glass microcapillary tubes (Drummond Scientific, Microcaps #1–0000100) and transported to the laboratory for analysis. The amount of cytokines in OSW from patients was measured using bead-based immunoassays for human cytokines (13-plex human cytokines/CVD magnetic bead panel; Millipore, #SPR1100) in the FLEXMAP 3D system (Luminex, MilliporeSigma, Burlington, MA). We used a custom-designed 13-plex human cytokine/CVD magnetic bead panel. We measured interleukin 10 (IL-10), interleukin-17A (IL-17A), interleukin-1 receptor antagonist (IL-1Ra), interleukin-1 alpha (IL-1α), neutrophil elastase (ELA2), interleukin-1 beta (IL-1β), tumor necrosis factor superfamily member 14 (LIGHT/TNFSF14), neutrophil gelatinase-associated lipocalin (NGAL), oncostatin M (OSM), interleukin 8 (IL-8), interferon gamma-induced protein 10, tumor necrosis factor alpha, and vascular endothelial growth factor cytokines. Sample volumes of 5 μL of OSW were loaded into a 96-well assay plate, and the assay was performed according to the manufacturer's instructions. The data were analyzed by using Belysa Immunoassay Curve Fitting software (MilliporeSigma, Burlington, MA). The absolute concentrations of the samples were determined by constructing a standard curve for each analyte.

For case report 2, the corneal epithelial thickness was measured using spectral-domain optical coherence tomography (OCT) with the RTVue XR 100 Avanti (Optovue, Inc, Fremont). Corneal In vivo confocal microscopy (IVCM) was performed on both eyes using the Heidelberg Retina Tomograph 3 with Rostock Cornea Module (HRT3 RCM; Heidelberg Engineering GmbH, Heidelberg, Germany).

### Data Analysis

Based on their subject groups as none oGVHD, probable oGVHD, and definite oGVHD, the clinical measurements (OSDI, Schirmer I test, corneal staining, and MMP-9 test score) and OSW cytokine measurements were combined for each group. The nonprogressed group comprised patients with none oGVHD at all post-HSCT visits. The progressed group included patients with either probable oGVHD or definite oGVHD at post-HSCT visits. The median with the interquartile range is reported for continuous variables. *P*-values for group comparisons at the subject or eye level were calculated using the nonparametric Kruskal–Wallis test for continuous variables. We summarized demographic data using the frequency with the percentage for categorical variables. All the statistical analyses were conducted using SPSS 25.0 software (IBM, Armonk, NY). Statistical significance was established for two-sided *P*-values below 0.05.

## RESULTS

This is a longitudinal study for determining the development of ocular GVHD after allogeneic hematopoietic stem cell transplant (HSCT) and a report of two cases that illustrate the “window of opportunity” concept in oGVHD treatment.

**FIG. 1.**
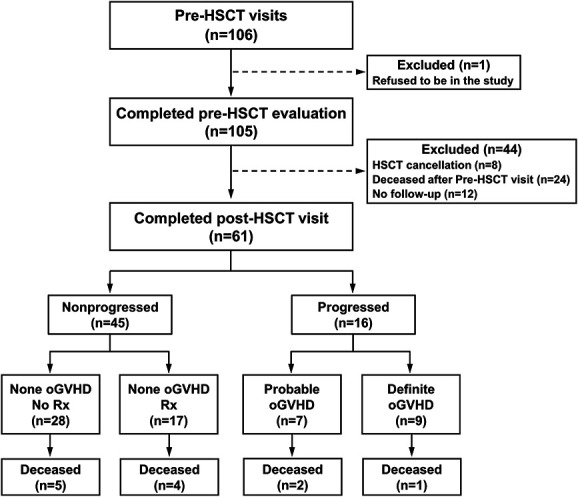
Longitudinal study flow diagram.

One hundred six patients consented to participate in the longitudinal study. One subject refused to complete the initial screening assessment and was excluded. Of the 105 patients enrolled, 44 patients did not return for any follow-up visits after undergoing the baseline visit. These patients included deceased (n=24), HSCT cancellation (n=8), and lost to follow-up (n=12). The remaining 61 patients were followed for up to 2 years post-HSCT (Fig. 1). Some patients missed scheduled follow-up visits. Clinical signs and symptoms were assessed at each follow-up visit, and eyes were graded based on the ICCGVHD classification system for the presence or absence of oGVHD. Progression from none oGVHD to probable or definite oGVHD was determined. Of the 61 patients, 26.2% (n=16; progressed group) developed either probable (11.5%, n=7) or definite oGVHD (14.8%, n=9) during the follow-up period, while 73.8% (n=45; nonprogressed group) did not develop oGVHD (none oGVHD) (Fig. 1). In the progressed oGVHD group, 62.5% of the patients developed systemic GVHD. By contrast, only 13% of the patients in nonprogressed group developed systemic GVHD. The demographics of the two groups (progressed and nonprogressed) are shown in Table 1.

**TABLE 1.** Demographic Data at the Baseline Visit (Pre-HSCT) of the Longitudinal Study

	Male	Female
Age (median [IQR])^*a*^	58 [44–66]	52 [35–62]
Gender (n, %)^*b*^	56 (53.3)	49 (46.7)
Race (n, %)^*b*^	56 (53.3)	49 (46.7)
White	36 (34.3)	36 (34.3)
Black or African American	7 (6.7)	9 (8.6)
Asian	5 (4.8)	0 (0.0)
Other/more than one	8 (7.6)	4 (3.8)
Ethnicity (n, %)^*b*^	56 (53.3)	49 (46.7)
Hispanic or Latino	21 (20.0)	13 (12.4)
Not Hispanic or Latino	33 (31.4)	36 (34.3)
Unknown	2 (1.9)	0 (0.0)
Indication (n, %)^*b*^	56 (53.3)	49 (46.7)
Leukemia	24 (22.9)	28 (26.7)
Lymphoma	9 (8.6)	4 (3.8)
Multiple myeloma	1 (1.0)	3 (2.9)
Myeloproliferative disorders	11 (10.5)	6 (5.7)
Other	11 (10.5)	8 (7.6)
Pre-HSCT visit (n, %)^*b*^	56 (53.3)	49 (46.7)
No post-HSCT visit (only came to pre-HSCT visit)^*b*^	23 (52.3)	21 (47.7)
Lost to follow-up visit after pre-HSCT	7 (15.9)	5 (11.4)
Dropped out of study	2 (4.5)	6 (13.6)
Deceased after pre-HSCT visit	14 (31.8)	10 (22.7)
Post-HSCT visit (n, %)^*b*^	33 (54.1)	28 (45.9)
Nonprogressed group	26 (42.6)	19 (31.1)
None oGVHD without treatment	18 (29.5)	10 (16.4)
None oGVHD with treatment	8 (13.1)	9 (14.8)
Progressed group	7 (11.5)	9 (14.8)
None oGVHD to probable oGVHD	3 (4.9)	4 (6.6)
None oGVHD to definite oGVHD	4 (6.6)	5 (8.2)
Deceased^*b*^	21 (58.3)	15 (41.7)
Deceased after pre-HSCT visit	14 (38.9)	10 (27.8)
Deceased during clinical study	7 (19.4)	5 (13.9)

^a,b^Median [25th, 75th] for continuous variables and frequency (%) for categorical variables.

HSCT, hematopoietic stem cell transplant; IQR, interquartile range; oGVHD, ocular graft-versus-host disease.

**FIG. 2.**
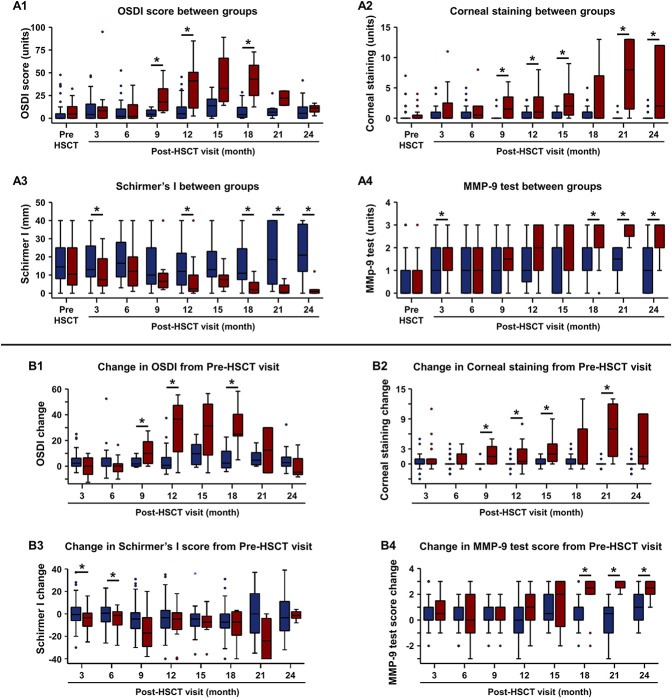
Comparison of clinical signs and symptoms between the nonprogressed and progressed groups at pre-HSCT and post-HSCT visits. Nonprogressed group: pre-HSCT (n=90 eyes), 3 months (n=70 eyes), 6 months (n=64 eyes), 9 months (n=26 eyes), 12 months (n=44 eyes), 15 months (n=14 eyes), 18 months (n=32 eyes), 21 months (n=14 eyes), and 24 months (n=26 eyes). Progressed group: pre-HSCT (n=32 eyes), 3 months (n=24 eyes), 6 months (n=22 eyes), 9 months (n=12 eyes), 12 months (n=16 eyes), 15 months (n=8 eyes), 18 months (n=10 eyes), 21 months (n=4 eyes), and 24 months (n=6 eyes). (A1–A4) Boxplots compare clinical signs and symptoms between groups at the pre-HSCT and each post-HSCT visit. (A1) OSDI score. (A2) Schirmer I test. (A3) Corneal staining. (A4) MMP-9 test. (B1–B4) Boxplots showing the comparison of the change in clinical signs and symptoms (post-HSCT visit minus pre-HSCT visit) between groups at each post-HSCT visit. Nonprogressed (blue), progressed (red). *P<0.05. HSCT, hematopoietic stem cell transplant; MMP-9, matrix metalloproteinase 9; OSDI, Ocular Surface Disease Index.

In the first analysis, we compared the median value of clinical signs and symptoms in the progressed and nonprogressed groups at pre-HSCT visits and each visit post-HSCT. The progressed group had greater severity of signs and symptoms than the nonprogressed group. The OSDI score in the progressed group was significantly greater than that in the nonprogressed group beginning from the 9-month visit and each subsequent visit after that. The OSDI at earlier clinic visits (3 and 6 months) was not significantly different (Fig. 2, A1). The OSDI in the progressed group at pre-HSCT visits (4.7 [0.0–14.8]) and post-HSCT visits of 3 months (8.4 [0.0–12.5]) and 6 months (2.1 [0.0–15.0]) did not show symptoms of ocular discomfort. The OSDI at 9 months showed mild symptoms of ocular discomfort (17.9 [7.8–37.5]). The OSDI in subsequent visits at 12 months (41.1 [8.1–53.0]), 15 months (32.9 [16.3–77.6]), and 18 months (42.9 [18.8–65.6]) showed severe symptoms of ocular discomfort. The corneal staining in the progressed group was significantly greater than that in the nonprogressed group at the 9-month visit and subsequent visits at 12 months and 15 months. The corneal staining at earlier clinic visits (3 and 6 months) was not significantly different (Fig. 2, A2). There was no corneal staining in the progressed group at pre-HSCT (0.0 [0.0–0.8]) and post-HSCT visit of 3 months (0.0 [0.0–2.8]). There was mild corneal staining at the 6-month visit (0.5 [0.0–2.0]). Corneal staining increased in subsequent visits at 9 months (1.5 [0.0–3.8]), 12 months (1.0 [0.0–3.8]), and 15 months (2.0 [0.3–4.0]). The Schirmer I values in the progressed group were significantly lower than those in the nonprogressed group at 3-month, 12-month, and 15-month visits. The Schirmer I values approached severe tear deficiency levels, defined as a value of ≤5 mm/5 min, at 9 months (6.5 [3.0–11.5]) and attained it at 12 months (2.5 [1.0–11.0]) (Fig. 2, A3). At 6 months, the Schirmer I values were normal (12.0 [4.0–20.8]). The matrix metalloproteinase 9 test score in the progressed group was significantly greater than that in the nonprogressed group at 3 months (Fig. 2, A4). During the pre-HSCT visit, 18 eyes (56%) in the progressed group had a negative MMP-9 test. Of these, 10 eyes (55.6%) turned MMP-9 positive or strong positive (MMP-9 score of ≥2) at 3-month and 6-month visits. By contrast, 46 eyes (51.1%) in the nonprogressed group had a negative MMP-9 test during the pre-HSCT visit, but only six eyes (13.0%) turned MMP-9 positive or strong positive at 3-month or 6-month visits.

In the second analysis, we determined the change in clinical signs and symptoms for each subject at post-HSCT visits from pre-HSCT visits and then compared the changes in signs and symptoms between the progressed and nonprogressed groups. The OSDI score in the progressed group was significantly greater than that in the nonprogressed group at 12-month and 18-month visits and approached significance (*P*=0.072) at 9-month visits. The OSDI change at earlier clinic visits (3 and 6 months) was not significantly different (Fig. 2, B1). Clinically significant OSDI change (OSDI>9.9) in the progressed group was seen at 9-month (10.0 [1.6–21.2]), 12-month (36.6 [8.1–50.0]), 15-month (31.3 [1.8–52.4]) and 18-month (25.0 [14.3–49.4]) visits. Clinically significant OSDI change was not seen in the nonprogressed group at any post-HSCT visits (Fig. 2, B1). The change in corneal staining in the progressed group was significantly greater than in the nonprogressed group at the 9-month visit and subsequent visits at 12 months and 15 months. The change in corneal staining at earlier clinic visits (3 and 6 months) was not significantly different (Fig. 2, B2). Clinically significant corneal staining value change (increase ≥1.0) in the progressed group was seen at a 9-month visit (1.5 [0.0–3.8]). Clinically significant corneal staining value change was not seen in the nonprogressed group at any post-HSCT visits. The change in Schirmer I values in the progressed group was significantly lower than that in the nonprogressed group at 6-month and 9-month visits. The change in the Schirmer I value at earlier clinic visits (3 months) was not significantly different. Clinically significant Schirmer I value change (reduction ≥10 mm) in the progressed group was seen at the 9-month visit (−17.0 [−29.5, −2.8]). Clinically significant Schirmer I value change was not seen in the nonprogressed group at any post-HSCT visits (Fig. 2, B3).

**FIG. 3.**
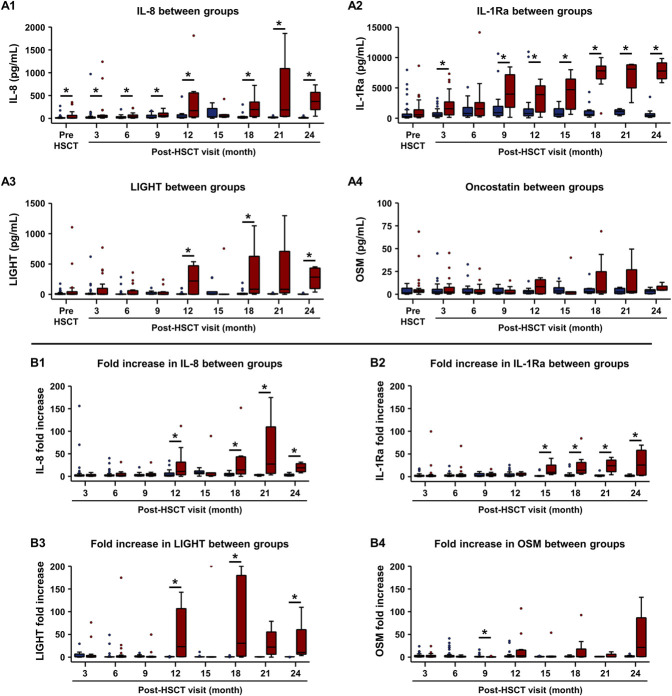
Comparison of inflammatory cytokines in OSW between the nonprogressed and progressed groups. Nonprogressed group: pre-HSCT (n=40 eyes), 3 months (n=32 eyes), 6 months (n=34 eyes), 9 months (n=20 eyes), 12 months (n=24 eyes), 15 months (n=10 eyes), 18 months (n=14 eyes), 21 months (n=6 eyes), and 24 months (n=12 eyes). Progressed group: pre-HSCT (n=30 eyes), 3 months (n=22 eyes), 6 months (n=20 eyes), 9 months (n=12 eyes), 12 months (n=12 eyes), 15 months (n=6 eyes), 18 months (n=8 eyes), 21 months (n=4 eyes), and 24 months (n=4 eyes). (A1–A4) Boxplots showing the comparison of OSW cytokines between groups at the pre-HSCT visit and each post-HSCT visit. (A1) IL-8, (A2) IL-1Ra, (A3) LIGHT, and (A4) OSM. (B1–B4) Boxplots showing the comparison of fold increase in OSW cytokines (post-HSCT visit divided by the pre-HSCT visit) between groups at each post-HSCT visit. (B1) IL-8, (B2) IL-1Ra, (B3) LIGHT, and (B4) OSM. Nonprogressed (blue), progressed (red). *P<0.05. HSCT, hematopoietic stem cell transplant; IL-8, interleukin 8; IL-1Ra, interleukin-1 receptor antagonist; OSM, Oncostatin M; OSW, ocular surface washings.

We also measured several cytokines in the OSW samples. In the first analysis, we compared OSW cytokine levels between the progressed and nonprogressed groups to determine significant differences. Interleukin 8 and IL-1Ra were significantly increased in the progressed group at seven out of eight post-HSCT visits, whereas all other cytokines were significantly increased in fewer than four visits (Fig. 3, A1–A4). At the 3-month visit, seven of 13 cytokines were significantly increased in the progressed group compared with the nonprogressed group. These cytokines include IL-1Ra, IL-8, IL-17A, ELA2, and NGAL. The second analysis determined the fold increase in OSW cytokine levels from pre-HSCT at each visit. We used a value of 2.0-fold increase (100% increase over baseline level) as a significant change (Fig. 3, B1–B4). At the 6-month visit, IL-8 (2.4-fold) and IL-1Ra (2.5-fold) were significantly increased in the progressed group. At the 9-month visit, IL-8 (3.4-fold), IL-1Ra (3.6-fold), ELA2 (2.6-fold), and NGAL (4.8-fold) were significantly increased. These cytokines remained significantly increased at all subsequent visits. In the nonprogressed group, IL-1Ra, but not any other cytokine, was significantly increased at 6-month (2.7-fold) and 9-month (2.5-fold) visits. Therefore, at the 6 to 9 months post-HSCT period that is considered high risk for oGVHD development, cytokines that have OSW levels more than 2.0-fold greater than pre-HSCT levels in the progressed group (but not in the nonprogressed group) include neutrophil-derived cytokines: IL-8, NGAL, and ELA2.

### Case Report 1

**FIG. 4.**
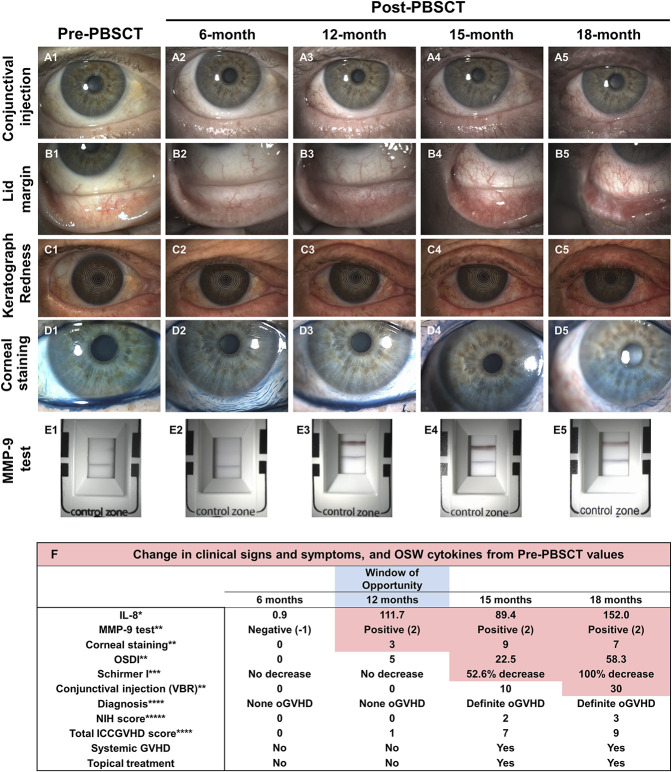
Case report 1: Allogeneic PBSCT was performed on a 76-year-old male patient diagnosed with primary idiopathic myelofibrosis. Images showing conjunctival injection (A1–A5), lid margin (B1–B5), Keratograph redness (C1–C5), corneal Lissamine green staining (D1–D5), and MMP-9 test (E1–E5) of the right eye at pre-PBSCT and post-PBSCT (6-, 12-, 15-, and 18-month) visits. (F) Detailed clinical signs and symptoms data and OSW at pre-PBSCT and post-PBSCT. *Fold change compared with baseline pre-HSCT levels; **Change in grade or score; ***Change in mm of filter paper wetting; ****Based on international consensus classification using absolute values; *****NIH consensus scoring classification. PBSCT, peripheral blood stem cell transplant; MMP-9, matrix metalloproteinase 9; NIH, National Institutes of Health; OSW, ocular surface washings.

A 76-year-old male patient diagnosed with primary idiopathic myelofibrosis underwent an Allogeneic Peripheral Blood Stem Cell Transplant (PBSCT) (Fig. 4). One month before PBSCT, an ophthalmological examination was performed to determine baseline clinical signs and symptom values. The patient was examined at prescheduled visits every 3 months post-PBSCT. At 3-month and 6-month post-PBSCT visits, clinical signs and symptoms were unchanged compared with the pre-PBSCT visit. MMP-9 test was negative, and IL-8 levels in OSW were not increased. At the 12-month post-PBSCT visit, the patient was asymptomatic (OSDI=5), and there was no change in the Schirmer I value; however, mild corneal staining was present. The international chronic oGVHD consensus group diagnostic criteria score was 1, corresponding to none oGVHD. The matrix metalloproteinase 9 test turned strong positive, and IL-8 levels in OSW increased more than 100-fold. At the 15-month visit, the patient had moderate symptoms of ocular discomfort (OSDI=22.5) and increased ocular bulbar redness. The Schirmer I value had reduced by 52.6%, showing a clinically significant ≥10 mm reduction. There was moderate corneal staining (9/15). The international chronic oGVHD consensus group diagnostic criteria score was 7, corresponding to the diagnosis of definite oGVHD. The MMP-9 test continued to be strong positive, and IL-8 levels in OSW were increased 90-fold. Combinatorial treatment was initiated with topical steroid eye drops, serum tears, and systemic doxycycline. At the 18-month visit, the clinical signs and symptoms worsened. The patient was severely symptomatic with the Schirmer I value of 0 mm. The international chronic oGVHD consensus group diagnostic criteria score was 9, corresponding to the diagnosis of definite oGVHD. In this patient, treatment was initiated at 15 months when the diagnosis of definite oGVHD was made; however, a “window of opportunity” existed at the 12-month visit when the diagnosis was still none oGVHD. Mild corneal staining and biomarker increase (MMP-9 and IL-8) were the only abnormalities during this “window of opportunity” for initiating treatment earlier.

### Case Report 2

**FIG. 5.**
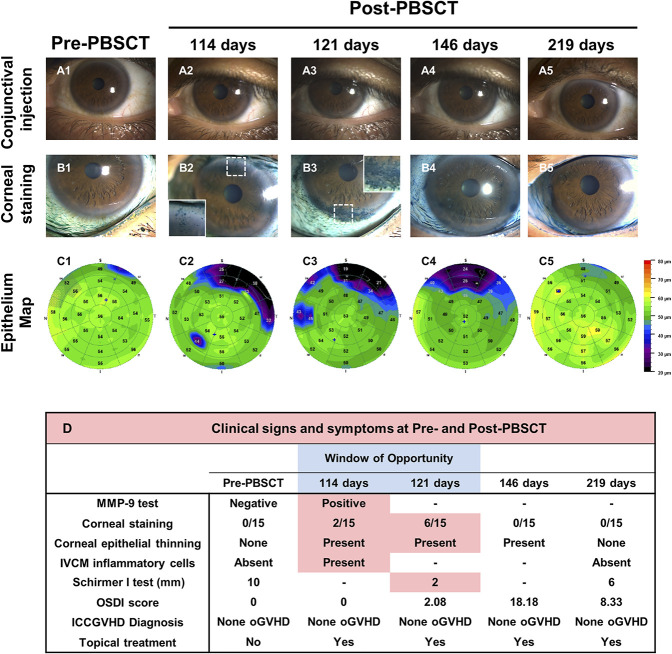
Case report 2: Allogeneic PBSCT was performed on a 55-year-old male patient diagnosed with MDS. Images showing conjunctival injection (A1–A5), corneal staining (B1–B5), and epithelium map (C1–C5) of the left eye at pre-PBSCT and post-PBSCT (114, 121, 146, and 219 days) visits. (D) Detailed clinical signs and symptoms at pre-PBSCT and post-PBSCT. MDS, myelodysplastic syndrome; PBSCT, peripheral blood stem cell transplant.

**FIG. 6.**
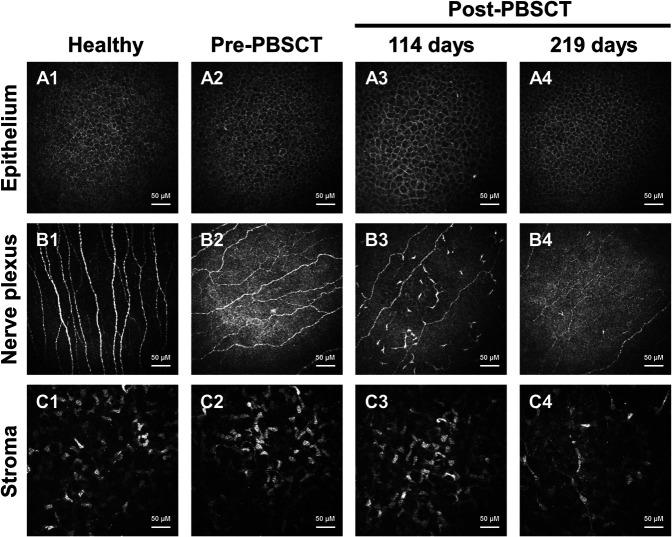
IVCM of case report 2, Left eye, central cornea. IVCM images show a healthy subject's epithelium (A1), sub-basal nerve plexus (B1), and anterior stroma (C1). IVCM of the patient in case report 2 showing epithelium (A2–A4), corneal sub-basal nerve plexus (B2–B4), and anterior stroma (C2–C4) of the central cornea of the left eye at pre-PBSCT and post-PBSCT (114 and 219 days) visits. IVCM, in vivo confocal microscopy, PBSCT, peripheral blood stem cell transplant.

A 55-year-old male patient diagnosed with myelodysplastic syndrome with excess blasts 2 underwent Allogeneic PBSCT (Figs. 5 and 6). Approximately 1 month before PBSCT, an ophthalmological examination was performed to determine baseline clinical signs and symptom values, and a clinic visit was scheduled for 3 months post-PBSCT. At the post-PBSCT visit (114 days post-PBSCT), the patient was asymptomatic (OSDI=0). There was no ocular bulbar redness. The matrix metalloproteinase 9 test was positive. Corneal epithelial OCT maps showed superior thinning. Corneal IVCM showed the presence of inflammatory cells at the sub-basal nerve plexus that had the appearance of “activated” dendritic cells. The patient was advised to start combinatorial topical treatment; however, he elected to use topical steroid eye drops only because he was asymptomatic and had no ocular redness. At the 121-day visit, the corneal staining had increased, and the Schirmer I values were now consistent with severe tear deficiency. The topical treatment was changed to combinatorial: methylprednisolone eye drops, serum tears, and pooled human immunoglobulin eye drops. At the 219-day visit, corneal staining had resolved entirely. The corneal epithelial thinning and the corneal inflammatory cells at the sub-basal nerve plexus were entirely resolved. The treatment was initiated in this patient based on the early sign of corneal staining and MMP-9 positivity in the “window of opportunity” while the diagnosis was still none oGVHD. This case report also demonstrates that the appearance of superior corneal epithelial thinning and inflammatory cells at the corneal sub-basal nerve plexus area may be additional signs for recognizing the “window of opportunity” and initiation of treatment.

## DISCUSSION

Our longitudinal study data suggest that tear fluid analysis for MMP-9 or neutrophil-derived cytokines (IL-8) may detect progression to ocular GVHD at earlier time points (3–6 months post-HSCT), as compared with clinical signs (corneal staining and Schirmer I test) and symptoms (OSDI). Clinically significant changes in signs and symptoms develop later (9 months post-HSCT). In the case report, corneal peripheral epithelial thinning developed earlier (4 months) while the patient was asymptomatic. Taken together, our data suggest that at 3 to 6 months post-HSCT, when the patient is still asymptomatic and has none oGVHD, an increase in the tear fluid cytokine and corneal epithelial thinning might be the only abnormalities. In the case reports, mild corneal staining was the earliest sign developing while the patient was asymptomatic. When symptoms and tear fluid deficiency become clinically significant, the patient may have progressed to probable or definite oGVHD. Therefore, a “window of opportunity” to initiate treatment exists before the progression of oGVHD. In our longitudinal study and case reports, the “window of opportunity” was identified by the following: (1) MMP-9 test converting from a negative pre-HSCT to a positive test post-HSCT; (2) several-fold increase in neutrophil-derived cytokines (e.g., IL-8) in the post-HSCT ocular surface wash as compared with pre-HSCT amounts; (3) appearance of peripheral corneal epithelial thinning as detected on post-HSCT OCT maps as compared with pre-HSCT; (4) appearance of corneal staining in corneas with no pre-HSCT staining; and (5) appearance of inflammatory cells (activated dendritic cells) at the sub-basal nerve plexus as detected by post-HSCT IVCM. Concurrent presence of two or more of these signs may be required for treatment initiation. Case report 1 demonstrates that the “window of opportunity” to initiate treatment was missed despite increases in tear fluid cytokines and early corneal staining. This patient progressed to definite oGVHD. Case report 2 demonstrates that the “window of opportunity” was identified by using a positive MMP-9 test, early corneal staining, corneal epithelial thinning, and inflammatory cells in the cornea. Prompt initiation of combinatorial topical treatment resulted in the resolution of the changes, and progression to oGVHD was preempted. The combinatorial topical therapy included the use of anti-inflammatory/immunosuppressive eye drop (steroid), immunomodulatory eye drop (pooled human immune globulin), and tear substitute eye drop (serum) that simultaneously targets several immunological pathways.^13^ In the longitudinal study, treatment modalities in the progressed group included topical eye drops (steroid eye drops, serum tears, erythromycin eye ointment, and artificial tears). In the nonprogressed group no treatment modalities were used other than artificial tears as needed. The progressed group responded with a reduction in signs and symptoms. While our data suggest that it might be possible to preempt the development of oGVHD during the “window of opportunity” with prompt combinatorial topical treatment, larger controlled multicentered studies are needed to support this management strategy fully.

The presence of each of the five signs that identify the “window of opportunity” has been previously reported in patients with dry eye disease and oGVHD. Corneal epithelial thinning has been reported in patients with dry eye disease, and in patients with limbal stem cell deficiency.^14,15^ Both conditions can occur in oGVHD.^16^ The peripheral epithelial thinning may represent an early sign due to inflammation-induced limbal stem cell stress. In case report 2, prompt initiation of combinatorial treatment reversed the epithelial thinning, which may have resulted from the resolution of limbal stem cell dysfunction. We and others have reported increased levels of IL-8 in the tear fluid of patients with oGVHD.^2,17,18^ Increased tear levels of IL-8 are significantly correlated with ocular surface parameters based on ICCGVHD criteria.^17^ IL-8 strongly correlates with fluorescein staining and has been proposed as a possible diagnostic marker of oGVHD.^18^ Tear fluid levels of MMP-9 also increase in oGVHD.^19^ In the clinical setting, MMP-9 presence in the tear fluid can be determined by using a commercially available point-of-care test (InflammaDry). Using this test, Giannaccare et al.^20^ reported that 62.8% of oGVHD eyes were strong-positive for MMP-9. By contrast, only 11.8% of eyes in the control group were weak-positive for MMP-9. The presence of inflammatory cells has been reported in the sub-basal nerve plexus area of the cornea using IVCM in patients with Sjögren syndrome as well as in those with non-Sjögren dry eye.^21,22^ We have reported the presence of inflammatory cells at the sub-basal nerve plexus area in patients with oGVHD.^23^ Levine et al.^24^ have reported that the presence of ≥2 activated dendritic cells (aDCs) in the central cornea suggests a systemic immune disorder in individuals with Dry Eye symptoms and that treatment with topical anti-inflammatory therapy can reduce the number of aDCs in the central cornea. The presence of long interdigitating dendrites or arms recognizes activated dendritic cells. In case report 2, the IVCM had shown greater than 2 aDCs that resolved after anti-inflammatory treatment.

Our longitudinal data and the case reports strongly argue against the overreliance on the development of symptoms of ocular discomfort for ophthalmological referrals and/or treatment initiation. When clinically significant ocular symptoms develop, progression to probable or definite oGVHD may have already occurred. Our data also make a strong argument for pre-HSCT Ophthalmological examinations and prescheduled post-HSCT Ophthalmological examinations (every 3–4 months post-HSCT) to detect changes associated with progression to oGVHD and early initiation of treatment. In the longitudinal study, 26.2% of the patients developed either probable or definite oGVHD. We analyzed the data regarding the initial ocular diagnosis of patients referred to our clinic by hematology/oncology services after HSCT. The reason for the referral to our clinic was new onset or worsening of dry eye symptoms. This referral pattern represents the current clinical practice favored by hematology/oncology services. Over the past 5 years, 65% of 375 such referred patients had probable or definite oGVHD at their initial visit to our clinic. The longitudinal study data and clinical referral experience inform us that scheduled follow-ups, including a pre-HSCT visit, result in fewer patients developing oGVHD (26%) as compared with waiting for post-HSCT patients to develop eye symptoms before referral for ophthalmological care. This may be because the majority of patients referred after the onset of eye symptoms will have already progressed to oGVHD by the time of their clinic visit; however, these data are not directly comparable. There is also a significant time lag between the decision by hematology/oncology services to refer to the post-HSCT patient and the ophthalmology clinical visit due to nonavailability of timely appointments. The longitudinal study data and our clinical practice experience also inform us that more than 40% of the patients who have a pre-HSCT ophthalmological exam do not have a single post-HSCT visit because of cancellation of HSCT or because of the deceased patient. Therefore, the recommendation of a routine pre-HSCT visit is likely to impose a burden on the patients, hematology/oncology services, and the ophthalmological services because approximately 50% of the patients will not have follow-ups. Additional clinical studies are needed to determine whether the 3-month post HSCT visit can substitute for the pre-HSCT visit for the purpose of providing baseline data and reducing the burden imposed by the pre-HSCT visit.

This study has several weaknesses. We could not ensure robust compliance with all follow-up visits for enrolled patients in the longitudinal study. Most of the patients were referred for participation in the study from other Chicago area hospitals, and these patients had to travel to UIC for study visits. This may have created an undue burden, as these patients were coping with their systemic illnesses and treatments. In addition, flare-ups of systemic chronic GVHD or hospitalizations due to infections or other complications interfered with our scheduled appointments, and rescheduling was not always feasible. This experience teaches that for efficient follow-ups after HSCT, the Ophthalmology service must be co-located with the hematology and oncology service, making ophthalmological appointments less burdensome. This study was conducted in a tertiary care specialty ophthalmological clinic with a singular focus on treating patients with dry eye and oGVHD. Our clinic houses equipment such as OCT, corneal IVCM, LipiView, and photo slit lamps, all of which may be needed to identify the “window of opportunity” for early treatment initiation. This level of expertise and equipment may not be available in all eye care practices, thus preventing wider adoption of clinical practice based on our findings. However, our results make a case for developing ophthalmology practices with advanced equipment and expertise in treating oGVHD to support hematology and oncology services in caring for these patients.

In conclusion, this report identifies clinical signs that may be used to determine a “window of opportunity” to recognize or predict the imminent onset of chronic oGVHD at an earlier stage before diagnostic criteria are met and before the patient complains of symptoms of ocular discomfort, thereby allowing for preemptive interventions to prevent progression to irreversible ocular surface damage. In addition, this report emphasizes the value of prescheduled clinic visits (pre-HSCT and every 3 months for 2 years post-HSCT).
